# Impact of male partner’s awareness and support for contraceptives on female intent to use contraceptives in southeast Nigeria

**DOI:** 10.1186/s12889-015-2216-1

**Published:** 2015-09-10

**Authors:** Echezona E. Ezeanolue, Juliet Iwelunmor, Ibitola Asaolu, Michael C. Obiefune, Chinenye O. Ezeanolue, Alice Osuji, Amaka G. Ogidi, Aaron T. Hunt, Dina Patel, Wei Yang, John E. Ehiri

**Affiliations:** Global Health and Implementation Science Initiatives, School of Community Health Sciences, University of Nevada, 4505 S. Maryland Pkwy, Box 453064, Las Vegas, 89154 NV USA; Department of Kinesiology and Community Health, University of Illinois, Urbana-Champaign, 123 Huff Hall, 1206S, Fourth St., Champaign, IL 61820 USA; Department of Health Promotion Sciences, Mel and Enid Zuckerman College of Public Health, University of Arizona, 1295 N. Martin Ave., Tucson, Arizona 85724 USA; Prevention, Education, Treatment, Training and Research-Global Solutions-PeTR-GS, Plot 25 Liberty Estate, Independence Layout Enugu, 400001 Enugu State, Nigeria; Healthy Sunrise Foundation, 8752 Castle Ridge Avenue, Las Vegas, NV 89129 USA; School of Community Health Sciences, University of Nevada, MS-274, RM212, Lombardi Recreation Center, Reno, NV 89557 USA

## Abstract

**Background:**

Despite the growing body of evidence on use of modern contraceptives among women in sub-Saharan African countries, little is known about the broader context in which female decision-making concerning contraceptive use occurs, particularly the role of their male partners’ awareness and support of modern contraceptives.

**Methods:**

We conducted a cross-sectional survey of 2468 pregnant women and their male partners enrolled in the Healthy Beginning Initiative (HBI), an intervention to increase HIV testing among pregnant women in Enugu, southeast Nigeria. The aims of this study were to determine: 1) male partners’ awareness of, and support for, female contraceptive methods, and 2) influence of male partners’ contraceptive awareness and support on pregnant women’s expressed desire to use contraception. We used logistic regression models to examine the association between male partners’ awareness and support of modern contraceptives on their spouses’ desire to use contraceptives.

**Results:**

Men’s awareness of, and support for, use of modern contraceptives were significantly associated with their female partners’ desire to use contraception. A majority of the men who were aware of modern contraceptives (66.5 %) and those who supported their spouses’ use of contraception (72.5 %) had partners who expressed a desire to use contraception. Men who were aware of female contraception were 3 times more likely to have spouses who desired to use contraception (AOR = 3.17, 95 % C.I: 2.70–3.75). In addition, men who showed support for their spouses’ use of contraception were over 5 times more likely to have spouses who indicated a desire to use contraception (AOR = 5.76, 95 % C.I: 4.82–6.88). Living in a household of 5 or more people (AOR = 1.45, 95 % C.I: 1.23–1.72) and residing in an urban area (AOR = 0.81, 95 % C.I: 0.67–0.97) were also significantly associated with women’s expressed desire to use modern contraception.

**Conclusion:**

Men’s awareness of, and support for, use of modern contraceptives were markedly associated with their spouses’ desire to use contraception. This underscores the need for men’s involvement in programs that seek to address women’s uptake of contraception in low and middle income countries.

## Background

Many women of reproductive age in sub-Saharan Africa do not use modern contraceptives due to various factors such as cost, side-effects, availability, influence of the extended family, and lack of spousal support [1–5]. In Ghana, Bawah et al. [2] observed that resource constraints which placed the purchase of contraceptive supplies in competition with buying basic necessities for family survival became all the more acute and stressful when male partners objected to fertility regulation. Previous studies conducted in Ghana and Nigeria suggest that spousal communication predicts contraceptive use [6, 7], and available evidence shows that women whose partners disapprove of modern contraceptive practice are unlikely to use them [6]. In Ethiopia, barriers to women’s unmet need for contraception include their husbands’ opposition, religion, poor knowledge, and lack of communication between spouses [8].

Although lack of family support, in particular, and several other barriers have been identified against women’s uptake of contraception in low and middle income countries, many family planning programs in these settings are designed with no consideration for the role of men in influencing their female partner’s contraceptive decision-making [8–11]. Some studies suggest that women who have greater autonomy and are involved in household decision-making are also able to make decisions related to their fertility [2, 12]. Also, changing gender norms, the influence of education, and increased awareness of the benefits of contraception among couples have been shown to influence spousal decisions regarding contraceptive uptake and continuation [2, 13, 14]. Nigeria has a high total fertility rate (TFR) estimated to be between 5.5 and 5.7 children per woman of reproductive age [1, 15, 16], with a low contraceptive use rate (15 %) among married women [15]. Understanding the role of men in their spouses’ contraceptive decision-making could contribute to efforts aimed at increasing uptake of contraception in Africa’s most populous nation. To provide insight into the broader context in which female contraceptive decision-making occurs in relation to the role of their male partners’ awareness and support of modern contraceptives, we analyzed data on a cross-sectional sample of 2468 pregnant women and their male partners who were enrolled in the Healthy Beginning Initiative (HBI), an intervention to increase HIV testing among pregnant women in southeast Nigeria. It is hoped that the resulting data will help to inform the planning of culturally appropriate interventions to address the significant unmet need for use of contraception among married women.

## Methods

### Study setting

This study is part of a cluster randomized trial that tested the comparative effectiveness of a congregation-based intervention to increase uptake of HIV counseling and testing among a cohort of church attending pregnant women and their partners in Enugu, southeast Nigeria [17].

### Study design and participants

This was a cross-sectional survey of 2468 pregnant women and their partners attending 40 churches in 40 communities, across 7 local government areas in Enugu State, southeast Nigeria. A final matched sample of 2393 men and their 2393 pregnant female partners with complete information was used for this analysis. The Healthy Beginnings Initiative (HBI) which provided the umbrella for this study was funded by the National Institutes of Health (NIH), and involved three steps: (a) church-organized prayer sessions for pregnant women that was used to recruit participants early in their pregnancy, (b) health education and integrated onsite laboratory tests (HIV, hepatitis B, and sickle cell genotype) implemented during church-organized baby showers to reduce stigma associated with HIV-only test, and (c) church-organized baby receptions, used for post-delivery follow-up and linkage to care [17]. Two women who were proficient in English and Ibo, the local language, were selected from each participating church and trained to serve as church-based health advisors (CHAs). They assisted with recruitment, informed consent procedures and questionnaire administration. The study was approved by the Institutional Review Board of the University of Nevada, Reno, and the Nigerian National Health Research Ethics Committee.

### Data collection

Survey instruments were administered to recruited pregnant women and their male partners by trained research assistants and CHAs. A complete list of all measures and details of the assessment protocol has been published elsewhere [17]. Male participants were asked the following questions among others: (a) are you aware of types of female contraceptive methods? (b) If yes, mention any methods that you are aware of. (c) Would you support your spouse’s use of any form of contraception (men’s support for contraception)? (d) If yes, what type? Female participants were asked the following: (a) are you aware of types of female contraceptive methods? (b) Are you interested in using any contraceptive method? (c) If yes, which type(s)? The survey instruments were pre-tested on a small sample of couples (10) who were not included in the main study.

### Statistical analysis

Chi-square and logistic regression models were used to determine the association between the outcome variable, women’s desire to use contraceptive, and predictor variables. Statistical significance was set at a p-value of less than 0.05. All analysis was performed using SAS 9.4 (Cary, North Carolina).

## Results

### Characteristics of participants

As shown in Table 1, more than half of the men who participated in this study (67.7 %) were aged 30 to 44 years. Less than half of the women (44.5 %) completed at least some secondary school education; a majority of the men full-time jobs (60.6 %), were Catholic (82.5 %), lived in rural areas (70.5 %) and had a household size of 4 or less individuals (55.2 %). More than half of the male partners were aware of female contraception (53.4 %) and supported use of contraception by their wives (55.3 %). About half of the women (54.1 %) interviewed expressed a desire to use contraceptives.

**Table 1:** Characteristics of respondents (Men)

	N	%
Age		
Less than 30	333	13.9
30 to 44	1619	67.7
45 to 59	412	17.2
60+	29	1.2
Education		
None	71	3.0
Primary	1005	39.0
Secondary	1065	44.5
Tertiary	323	13.5
Spouse (Wife)’s Educational level		
None	29	1.2
Primary	561	23.4
Secondary	1366	57.1
Tertiary	437	18.3
Employment		
Full Time	1450	60.6
Part Time	546	22.8
Unemployed	397	16.6
Aware of Female Contraception		
Yes	1279	53.4
No	1114	46.6
Support spouse’s use of contraception		
Yes	1323	55.3
No	1070	44.7
Spouse (wife) interested in contraceptive		
Yes	1294	54.1
No	1099	45.9
Household Characteristics		
Church		
Anglican	420	17.5
Catholic	1973	82.5
Area of Residence		
Rural	1687	70.5
Urban	706	29.5
Household Size		
4 or Less	1320	55.2
5 or more	1073	44.8
TOTAL	2393	100

### Men’s socio-demographic characteristics and support for their spouse use of for contraceptives

As shown in Table 2, men’s awareness of, and support for, use of contraception were significantly associated with their spouses’ desire to use contraceptive; 66.5 % of men who demonstrated awareness of a modern female contraceptive method, and 72.5 % who supported their partner’s use of contraceptive had partners who desired contraception. Household characteristics such as area of residence and household size were also associated with women’s desire to use contraception. Fifty per cent of the men who lived in urban areas and 58.6 % of those with household size of 5 or more people had partners who expressed a desire to use contraception.

**Table 2:** Men’s socio-demographic characteristics and support for their spouse’s use of contraceptives

	Female partners’ desire to use contraceptive (yes vs. no)			
	No	Yes	Crude OR^a^	Adjusted OR
	N (%)	N (%)	(95 % CI)	(95 % CI)
Aware of Female Contraception^*^				
Yes	428 (33.5)	851 (66.5)	3.01 (2.55–3.56)	3.17 (2.70–3.75)
No	671 (60.2)	443 (39.8)	REF	REF
Support spouse’s use of contraception^*^				
Yes	364 (27.5)	959 (72.5)	5.78 (4.84–6.90)	5.76 (4.82–6.88)
No	735 (68.7)	335 (31.3)	REF	REF
Demographic Characteristics				
Church				
Catholic	913 (46.3)	1060 (53.7)	0.92 (0.75–1.14)	0.91 (0.73–1.12)
Anglican	186 (44.3)	234 (55.7)	REF	REF
Area of Residence^**^				
Urban	353 (50.0)	353 (50.0)	0.79 (0.67–0.95)	0.81 (0.67–0.97)
Rural	746 (44.2)	941 (55.8)	REF	REF
Household Size^**^				
4 or Less	655 (49.6)	665 (50.4)	REF	REF
5 or more	444 (41.4)	629 (58.6)	1.40 (1.19–1.64)	1.45 (1.23–1.72)
Age				
Less than 30	147 (44.1)	186 (55.9)	REF	
30 to 44	731 (45.2)	888 (54.8)	0.96 (0.76–1.22)	
45 to 59	206 (50.0)	206 (50.0)	0.79 (0.59–1.06)	
60+	15 (51.7)	14 (48.3)	0.74(0.35–1.58)	
Spouse (Wife)’s Educational level				
None	13 (44.8)	16 (55.2)	REF	
Primary	270 (48.1)	291 (51.9)	0.88 (0.41–1.85)	
Secondary	596 (43.6)	770 (56.4)	1.05 (0.50–2.20)	
Tertiary	220 (50.3)	217 (49.7)	0.80 (0.38–1.71)	
Men’s Education				
None	24 (33.8)	47 (66.2)	REF	
None/Primary	419 (44.9)	515 (55.1)	0.63 (0.38–1.04)	
Secondary	492 (46.2)	573 (53.8)	0.60 (0.36–0.99)	
Tertiary	164 (50.8)	159 (49.2)	0.50 (0.30–0.85)	
Employment				
Unemployed	192 (48.4)	205 (51.6)	REF	
Full Time	660 (45.5)	790 (54.5)	1.12 (0.90–1.40)	
Part Time	247 (45.2)	299 (54.8)	1.13 (0.88–1.47)	

^a^Adjusted Model controlled for men’s age, education, and employment status

^b^Statistical significance (*p*-value) reported for bivariate analyses: ^*^ and ^**^ represent *p <*0.0001 and *p <* 0.05 respectively

After adjusting for men’s age, education, and employment status, logistic regression models showed that men’s awareness of, and support for, female contraception were significantly associated with women’s expressed desire to use contraception. Men who were aware of female contraception were significantly more likely (AOR = 3.17, 95 % C.I: 2.70–3.75) to have spouses who expressed a desire to use contraception. Similarly, men who showed support for their spouses’ use of contraception were significantly more likely (AOR = 5.76, 95 % C.I: 4.82–6.88)] to have spouses who expressed a desire to use contraception. On the other hand, residing in an urban area (AOR = 0.81, 95 % C.I: 0.67–0.97) had a negative association with women’s desire to use contraception while men living in a household of 5 or more people (AOR = 1.45, 95 % C.I: 1.23–1.72) tended to have partners who expressed a desire to use contraception.

## Discussion

Since Nigeria is the most populous country in sub-Saharan Africa, with an estimated population of over 170 million people [18], use of contraceptives is increasingly important given the substantial level of unmet need for family planning within the country [15]. While previous studies on contraceptive uptake focused on women or men independently [9], our study examined pregnant women and their male partners as a dyad to identify the extent to which women’s desires to use contraception were linked to their male partners’ awareness and support of contraceptives.

The results of our analysis highlight two key conclusions that merit further attention. First, we found that men’s awareness of, and support for, modern contraceptives were largely associated with their spouse’s desire to use contraception. This finding is supported by the cultural norm in Nigeria and indeed much of sub-Saharan Africa where men have important and often dominant role in fertility decisions [2, 9, 19, 20]. It also suggests that men may potentially have more decision-making power with the actual behavior concerning contraceptive use [2, 9]. While this finding does not imply a causal relationship due to the nature of the study’s cross-sectional design, studies conducted in other low and middle income countries have found that decision-making concerning contraceptive use among couples was determined largely by the male partners’ desire for more children [8]. Second, when socio-demographic characteristics of participants were examined further, we found that household size was more important in influencing spousal desire to use contraceptives than area of residence (urban versus rural). This finding supports existing literature which shows a relationship between having more children and an increased desire for contraception [12].

We found no significant associations between most individual level variables considered in this study. For example, the analyses did not demonstrate marked associations between men’s age, or employment and women’s desire to use contraception. This finding suggests that use of modern contraceptives may be influenced by other factors including community level or system level factors such as social/community networks [21–24] or the influence of mass media [13, 25, 26]. Indeed, previous studies have demonstrated a positive influence of mass media on awareness, support, and decision making concerning uptake and use of contraceptives [26]. Future research on contraceptive perceptions, awareness, and support will benefit from moving beyond individual-level variables to consider how community level factors, such as the role of social networks or the presence of specific diffusion effects such as mass media, influence uptake of contraceptives in low and middle income countries.

### Limitations

While the analyses revealed an association between men’s knowledge of, and support for contraception and their spouses’ use of contraception, it is important to acknowledge that knowledge and support may not translate to actual behaviors that may be influenced by cultural and societal norms [27]. Thus, improving women’s use of contraceptives will benefit from future studies that explore reasons why men’s awareness of, and support for, contraception may not translate to actual contraceptive use by their spouses. Furthermore, our analyses did not examine other factors such as the role of social networks [24] or the influence of mass media [26] in diffusing messages on contraceptive uptake and use. Equally, although the primary HBI study within which this study was embedded used a cluster randomized design, collection of data on men’s support for their spouses’ use of contraceptives and pregnant women’s expressed desire to use contraception was done using a cross-sectional survey involving all HBI participants. For confidentiality reasons, we did not include identifiers that could be used to link participants’ to specific clusters. Thus, we were unable to conduct hierarchical analysis to assess whether results were different or similar across clusters and individual participants. Finally, because of the cross-sectional nature of our data, we were unable to determine any causal relationships between the variables examined. Longitudinal studies will be necessary to establish causal relationships between men’s awareness of, and support for, contraception in relation to use of contraceptives by their spouses, and other variables that influence this relationship.

## Conclusion

The consistent finding regarding the influence of men on contraceptive use desire by their spouses suggests that their inclusion in family planning programs in low and middle income countries is crucial for success [9–11]. Indeed, focusing on men’s attitudes could potentially increase the opportunity to: 1) explore ways to increase uptake and continuation of family planning methods; 2) increase the proportion of pregnancies that are intended; 3) reduce maternal and infant morbidity and mortality associated with unintended pregnancy; and 4) prevent maternal-to-child transmission of HIV while also improving health outcomes of women of reproductive age. Similarly, programs that seek to increase uptake of contraceptive services by married women in Nigeria and other countries in sub Saharan Africa should include efforts to understand how individual and community level factors influence men and women’s attitudes and behavior towards contraception.
